# A hepatic sclerosed hemangioma with significant morphological change over a period of 10 years: a case report

**DOI:** 10.1186/1752-1947-7-139

**Published:** 2013-05-28

**Authors:** Yoshitaka Shimada, Yoshihito Takahashi, Hiroyoshi Iguchi, Hitoshi Yamazaki, Hidekazu Tsunoda, Masaaki Watanabe, Masaya Oda, Hiroaki Yokomori

**Affiliations:** 1 Department of Internal Medicine, Kitasato University Medical Center, 6-100 Arai, Kitamoto-shi, Saitama 364-8501, Japan; 2 Department of Surgery, Kitasato University Medical Center, Saitama, Japan; 3 Department of Radiology, Kitasato University Medical Center, Saitama, Japan; 4 Department of Pathology, Kitasato University Medical Center, Saitama, Japan; 5 Organized Center of Clinical Medicine, International University of Health and Welfare, Tokyo, Japan

**Keywords:** Caveolae, Caveolin-1, Electron microscopy, Hepatic sclerosed hemangioma, Immunohistochemistry, Liver cavernous hemangioma

## Abstract

**Introduction:**

Liver cavernous hemangioma is the most common noncystic hepatic lesion, and a hemangioma that undergoes degeneration and fibrous replacement is called a hepatic sclerosed hemangioma.

**Case presentation:**

A 63-year-old Japanese man was admitted for detailed investigation of a liver tumor. Tumor markers carcinoembryonic antigen, alpha-fetoprotein, and CA19-9 levels in the peripheral blood were not elevated at any time. Plain computed tomography showed an approximately 1.5cm low density mass in the periphery of segment 8, which was marginally enhanced on contrast-enhanced dynamic computed tomography. On magnetic resonance imaging, the tumor was hypointense on T1-weighted image and hyperintense on T2-weighted image. The tumor was suspected to be an atypical hemangioma, metastatic, hepatocellular carcinoma, or cholangiocellular carcinoma. Segmental hepatectomy was performed. Histological examination of the resected tumor specimen revealed a sclerosed hemangioma with marked hyalinization and sparse stromal fibrosis. Immunochemically, the tumor cells were positive for CD34 and alpha smooth muscle actin. Electron microscopically, the residual hemangioma consisted of numerous caveolae and vesicles in endothelial cells in irregular shapes and sizes. Immunostaining for caveolin-1 showed decreased or no caveolin-1 reactivity in the hyalinized lesions of the sclerosed hemangioma, but abundant caveolin-1 reactivity in the residual cavernous hemangioma. Of interest, computed tomography images of the tumor obtained 10 years earlier at our hospital depicted a 3cm typical cavernous hemangioma.

**Conclusions:**

Hepatic sclerosed hemangioma is a rare condition. Comparison of radiological findings of the lesion over a period of 10 years was valuable in providing insight for the evolutional process from liver cavernous hemangioma to hepatic sclerosed hemangioma.

## Introduction

Liver cavernous hemangioma (LCH) is the most common noncystic hepatic lesion, with a reported incidence of 20% of benign hepatic tumors [1]. Most hemangiomas remain stable on follow-up imaging [2]; growth and spontaneous regression are reported to be very rare [3]. LCH that have undergone degeneration and fibrous replacement are called sclerosed, thrombosed, or hyalinized hemangiomas [4]. On the one hand, Shepherd and Lee [5] first mentioned hepatic sclerosed hemangioma (HSH) as a differential diagnosis of solitary necrotic nodules in addition to a list of benign lesions including traumatic etiology, a sequel of previous infection, and LCH. On the other hand, Berry [6] suggested that solitary necrotic nodules might all be HSH.

Computed tomography (CT) features suggestive of HSH include geographic outline, capsular retraction, decrease in size over time, and loss of previously observed regions of enhancement. Additional features include the presence of transient hepatic attenuation difference, rim enhancement, and nodular regions of intense enhancement as seen in typical hemangiomas [7]. The common histological features of HSH comprise multiple thin walled vessels within a hypocellular stroma demonstrating varying degrees of fibrosis and sclerosis. Macroscopically, a hemangioma is usually reddish-blue and well demarcated from surrounding tissue. However, the sclerosed variant is seen as a pale nodule if there is significant fibrosis present. The cell type of origin is mesenchymal and as such the lesions can occur almost anywhere [8]. Makhlouf and Ishak [9] suggested that mast cells play a pivotal role in the development of HSH, perhaps representing a distinct histological subtype of liver lesion. In the present case, radiological images obtained 10 years earlier were available for comparison, which were valuable in providing insights to the evolutional process from LCH to HSH.

Caveolae are cell plasma membrane microdomains and are responsible for transmembrane trafficking, endocytosis and lipid homeostasis, and also serve in signaling processes as a compartment where receptors and signaling proteins are concentrated [10]. Caveolin-1 in endothelial cells regulates angiogenesis, microvascular permeability and vascular remodeling [10,11]. Apart from its signaling function in normal cells, caveolin-1 also functions as a tumor suppressor and pro-apoptotic protein [12]. Previous study has indicated that this protein is overexpressed in different cancers and that it might serve as a prognostic factor for patient outcome or contribute to metastatic spread [13]. Caveolin expression has been described in a range of vascular neoplasms including lobular capillary hemangiomas, targetoid hemosiderotic hemangiomas, and tufted angiomas, and decreased expression of this protein was detected in angiosarcomas, Kaposi’s sarcoma, and epithelioid hemangioendotheliomas [14]. These findings suggest that anti-caveolin antibodies may play a useful role in distinguishing benign from malignant vascular neoplasms. In the present case, we investigated caveolin-1 expression of HSH by immunohistochemistry.

## Case presentation

**Figure 1:**
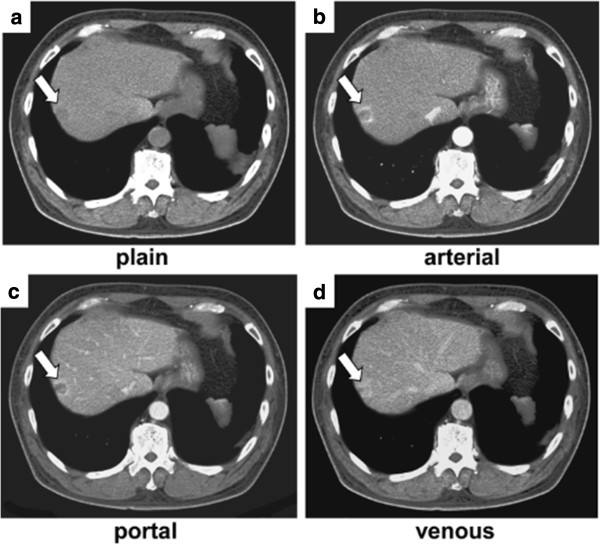
**Abdominal contrast-enhanced dynamic computed tomography (CT) findings. a**: Plain CT shows an approximately 1.5cm low-density mass in the periphery of segment 8 (arrow). **b**: Early arterial phase image (40sec) of enhanced CT shows that the tumor is marginally enhanced. Small satellite-like lesions (arrow) are found close to the main tumor. **c**: Portal venous phase image of enhanced CT shows the tumor in segment 8 (arrow). **d**: Delayed-phase image (180 seconds) of enhanced CT shows faint enhancement of a small portion of the tumor. Arrow denotes hepatic mass lesion.

**Figure 2:**
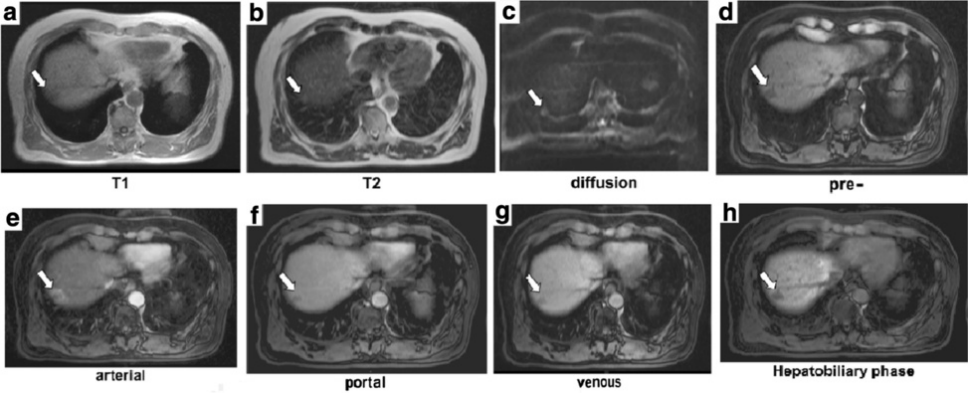
**Magnetic resonance imaging findings. a**: T1-weighted image demonstrates the tumor as a hypointense area with satellite-like lesions in segment 8 (arrow). **b**: T2-weighted image shows the main tumor as a slight hyperintense area (arrow). **c**: A diffusion-weighted image depicts the main tumor as a slightly hyperintense area (arrow). **d-h**: Dynamic gadolinium ethoxybenzyl diethylenetriaminepentaacetic acid-enhanced magnetic resonance imaging of the tumor (arrow).

A 63-year-old Japanese man was transferred to our hospital for detailed investigation of a liver tumor. At admission to our hospital, the hematologic and blood chemistry data were as follows: white blood cell count 3,800/μL, hemoglobin 15.9g/dL, platelet count 146,000/μL, blood urea nitrogen 17.9mg/dL, creatinine 0.82mg/dL, albumin 4.3g/dL, aspartate aminotransferase 26IU/L, alanine aminotransferase 33IU/L, and total bilirubin 0.9mg/dL. Serum hepatitis B (HB) surface antigen, anti-HBe antibody, and anti-hepatitis C virus antibody were negative. Alpha-fetoprotein, carcinoembryonic antigen, and cancer antigen 19–9 levels were within the normal ranges. Dynamic CT depicted a mass measuring 1.5 × 1.4cm with a bulging contour in segment 8 of the liver, which was enhanced in the arterial phase and showed subtle low density with focal capsular enhancement in the delayed phase (Figure 1). A magnetic resonance image (MRI) demonstrated low signal intensity on T1-weighted image, and slightly high signal intensity on T2-weighted and diffusion-weighted images (Figure 2a–c). Dynamic contrast (gadolinium ethoxybenzyl diethylenetriaminepentaacetic acid)-enhanced MRI of the tumor showed subtle marginal enhancement in the delayed phase and well-demarcated low intensity in the hepatobiliary phase (Figure 2d-h). Gastroscopy revealed atrophic gastritis and colonoscopy showed normal findings.

**Figure 3:**
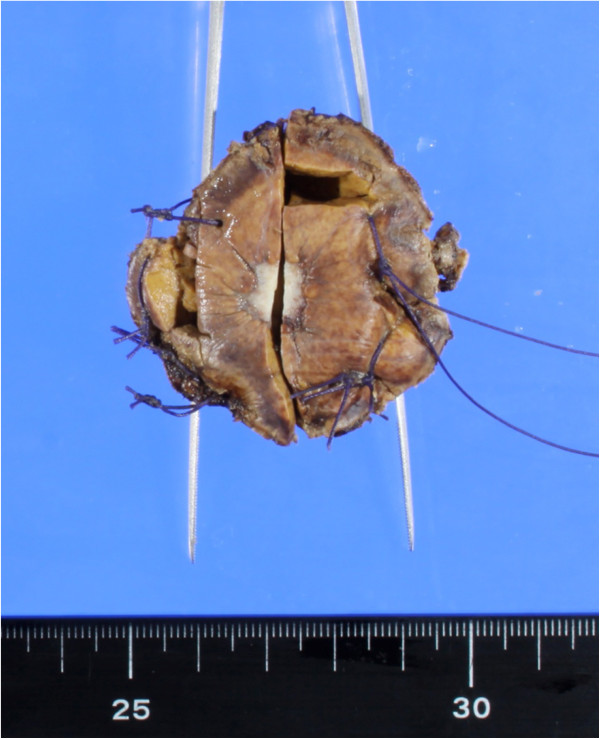
**Gross appearance of the sclerosed hemangioma.** The surface discloses a well-demarcated homogenous gray-white solid nodule, measuring 1cm at its greatest dimension.

**Figure 4:**
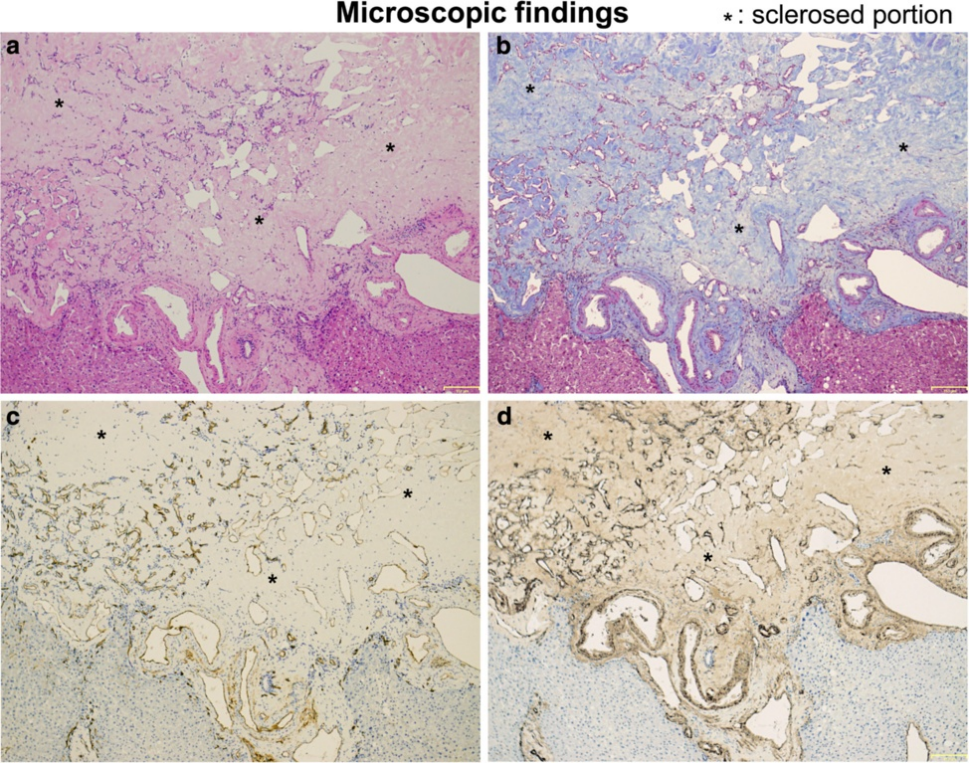
**Histologic features of the sclerosed hemangioma nodule. a**: A hyalinized mass is distinguished from the surrounding normal liver tissue (hematoxylin and eosin staining). **b**:The mass is composed of collapsed vascular spaces on a rich paucicellular fibrous stroma. Sclerosis is highlighted by Masson’s trichrome stain. **c**: The vascular spaces are clearly identified by immunohistochemistry for the endothelial marker CD34. **d**: The vascular and cavernous hemangioma spaces are identified by immunohistochemistry for the vascular smooth muscle cell marker, alpha smooth muscle actin. Asterisks denote sclerosed portion.

Based on the radiologic findings, we suspected the tumor to be an atypical hemangioma, metastatic, hepatocellular carcinoma, or a cholangiocellular carcinoma. Due to a suspicion of carcinoma, a hepatic segmentectomy of segment 8 was performed. On pathological examination, the resected liver tumor measured 1.1cm × 1.1cm × 1.0cm. Sectioning revealed a relatively homogenous, well-circumscribed white solid nodule with several dark-red, pin-point spots (Figure 3). Histopathological evaluation revealed that most areas were composed of sclerotic hyalinized collagenous tissues with scattered tiny-to-small, thin-walled vascular spaces (Figure 4a and b). The vascular spaces were frequently collapsed and lined by flat endothelial cells. Immunohistochemical studies showed that the cells were positive for CD34, an endothelial marker (Figure 4c) and alpha smooth muscle actin, a marker of vascular smooth muscle (Figure 4d). Based on these pathological findings, the tumor was diagnosed as HSH.

**Figure 5:**
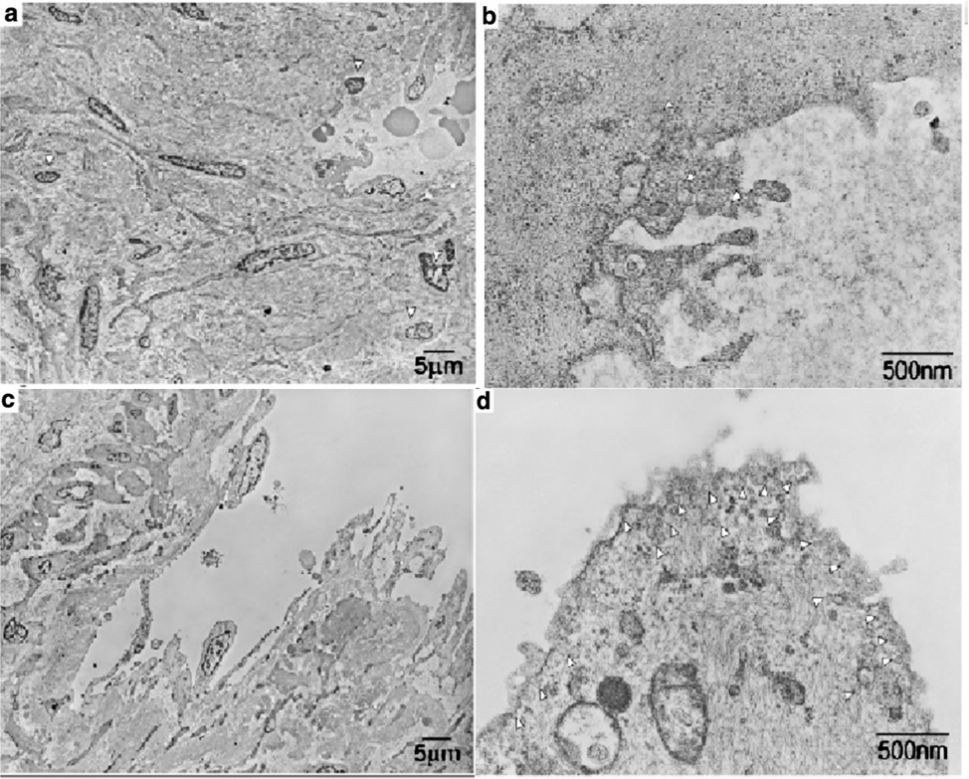
**Electron microscopic findings. a**: Electron micrograph shows a hyalinized lesion. Cells resembling histiocytes, fibroblast-like cells, and remnant endothelial cells derived from sclerosed hemangioma are observed. White arrowheads denote histiocytes or mast cells. Bar denotes 5μm. **b**: At high magnification, a remnant endothelial cell contains a few micropinocytic vesicles and caveolae, but numerous cytoplasmic filaments. White arrowheads denote caveolae. Bar denotes 500nm. **c**: Residual hemangioma is composed of numerous caverns in various shapes and sizes. The caverns form a labyrinth, communicating with each other. They are lined by spindle-shaped endothelial cells. Bar denotes 5μm. **d**: At high magnification, residual endothelial cells are composed of many micropinocytic vesicles or caveolae on the luminal surface. Residual cavernous hemangioma contains numerous cytoplasmic filaments. White arrowheads denote caveolae and vesicles. Bar denotes 500nm. Uranyl acetate and lead citrate staining.

**Figure 6:**
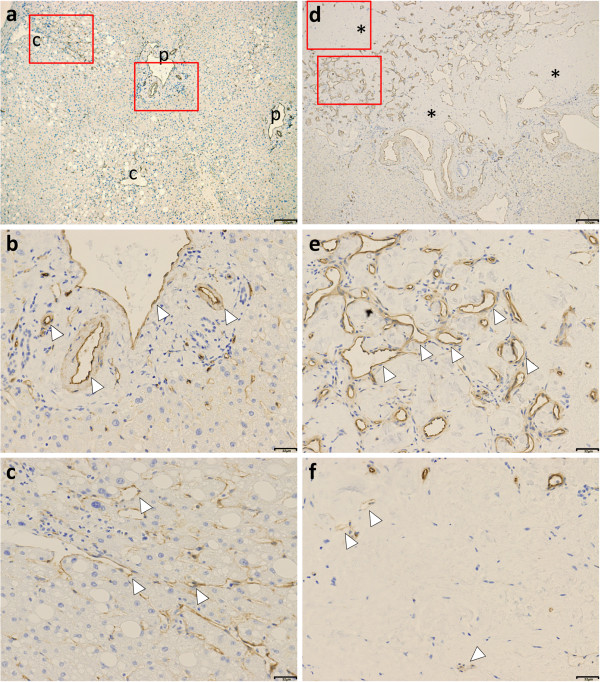
**Immunostaining for caveolin-1 in normal liver areas (a-c) and the lesion (d-f).** For the lesion, immunostaining was performed on serial sections continuous with those used in Figure 4. **a-c**: normal liver areas **a**: Abundant caveolin-1 reactivities are seen in both the endothelial cells as well as smooth muscle cells in normal areas. P denotes portal tract. C denotes central vein. Bar denotes 154μm. **b**: Caveolin-1 reactivities are noted on the hepatic artery, capillary venules, and portal vein in the portal tract in normal control liver areas. Arrow heads denote hepatic artery, capillary venules, and portal vein. Bar denotes 32μm. **c**: Caveolin-1 reactivities are detected in the hepatic sinusoidal lining cells around pericentral zone 3 in normal control liver areas. Arrowheads denotes liver sinusoidal lining cell. Bar denotes 32μm. **d-f**: lesion. **d**: Caveolin-1 reactivities are reduced or absent in the hyalinized lesions of sclerosed hemangioma. In residual hemangioma, high expression of caveolin 1 is found in endothelial cells. Caveolin-1 reactivity is almost absent in fibroblasts. Asterisks denote sclerosed portion. Bar denotes 153μm. **e**: Lesion of liver cavernous hemangioma lesion. Arrowhead denotes endothelial cells. Bar denotes 32μm. **f**: Lesion of sclerosed hepatic hemangioma. Arrowhead denotes endothelial cell. Bar denotes 32μm. Red rectangles denote the regions seen in 6b or 6c and 6e or 6f, respectively.

Furthermore, we also observed the tumor by electron microscopy and investigated the expression of caveolin-1 by immunohistochemistry (Additional file 1). On electron micrograph, the tumor appeared to be hyalinized. Cells resembling mast cells or histiocytes, fibroblast-like cells, and remnant endothelial cells were observed (Figure 5a). Remnant endothelial cells contained a few micropinocytic vesicles and caveolae, but numerous cytoplasmic filaments (Figure 5b). We also found residual LCH composed of numerous caverns in various shapes and sizes. The caverns formed a labyrinth, communicating with each other. They were lined by spindle-shaped endothelial cells (Figure 5c). The endothelial cells also contained numerous cytoplasmic filaments (Figure 5d). Moreover, caveolae and multiple micropinocytic vesicles were observed along the luminal and basal cell surfaces. Slender intraluminal processes were also found, sometimes overlapping with the cytoplasmic extensions from other cells (Figure 5c and d). Immunohistochemistry revealed caveolin-1 expression on the hepatic artery, capillary venules, portal vein in the portal tract, and in the hepatic sinusoidal lining cells around pericentral zone 3 in normal control liver areas (Figure 6a-c). Caveolin-1 remained overexpressed in the endothelial cells of the capillary tufts at the edge of the residual LCH but was reduced in the sclerosed hyaluronic lesion (Figure 6d-f). High expression of caveolin-1 was observed in the endothelial cells of the hemangioma (Figure 6e). Caveolin-1 immunostaining was nearly absent in fibroblasts (Figure 6f).

**Figure 7:**
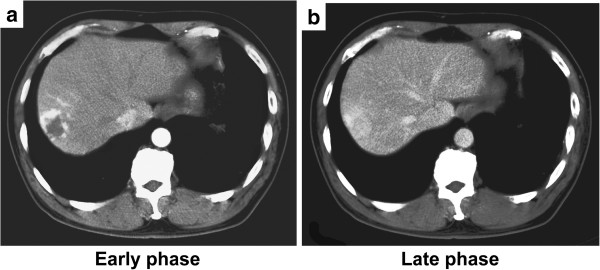
**Two-phase dynamic incremental computed tomography (CT) of the lesion performed 10 years ago. a**: In the early phase CT image, the lesion in segment 8 shows peripheral high attenuation. **b**: The late phase CT image demonstrates homogeneous high attenuation. These images demonstrate the progressive, centripetal contrast enhancement in a liver cavernous hemangioma.

CT images obtained from the same patient 10 years ago were available, and provided insight for the evolutional process from LCH (showing peripheral high attenuation in the early phase and homogeneous high attenuation in the late phase; Figure 7a and b) to HSH (showing peripheral high density in the arterial phase and subtle low density with focal capsular enhancement in the delayed phase; Figure 1).

The postoperative course was uneventful and the patient was discharged after 2 weeks.

## Discussion

HSH is a rare condition. In the present case, we investigated the HSH using electron microscopy and immunohistochemical methods. LCH are usually found incidentally and are readily diagnosed from their characteristic homogeneous hyperechogenicity and posterior acoustic enhancement on ultrasound examination. They may sometimes present different stages of evolution. In these cases, radiological findings show atypical features, occasionally mimicking malignant lesions [15]. Although not pathognomonic, some features of HSH may suggest a presumptive diagnosis and lead to biopsy rather than more extensive intervention [7]. Due to a suspicion of carcinoma, we performed surgical segmental resection in the present case. Some pathological changes are responsible for the variations of the radiological findings of hemangioma. Of these complications, sclerosing hemangiomas may have a variable amount of stroma, varying from scanty (fibrillar or hyaline) to abundant (hyaline or sclerotic), and sclerosed hemangiomas are characterized by extensive fibrosis with subsequent hyalinization and marked narrowing or obliteration of the vascular spaces [16]. Makhlouf and Ishak [9] reported distinct clinical and histopathological differences between sclerosing cavernous hemangioma and HSH, and suggested that recent hemorrhage, hemosiderin deposits, and abundant mast cells are present in sclerosing hemangioma. In our case, HSH was presumptively diagnosed from light microscopic observations of extensive fibrosis with hyalinization and marked narrowing or obliteration of the vascular spaces. Makhlouf and Ishak [9] also reported the possible involvement of mast cells in angiogenesis, the regression process and the development of fibrosis. According to their report, sclerosing hemangiomas show immunopositivity more frequently for collagen IV, laminin, factor VIII-R antigen, CD34 and CD31, as well as increased immunoreactivity for smooth muscle actin when compared with sclerosed hemangiomas. Moreover, fibrosis, increased elastic fibers, and dystrophic or psammomatous calcifications with a decreased number of mast cells can be observed in the sclerosed hemangioma [9]. In our present case, HSH was definitively diagnosed based on routine hematoxylin and eosin and Masson trichrome staining, as well as CD34 immunostaining (Figure 4).

In the present case, a CT showed an heterogeneous enhancement at the marginal portion of the tumor. MRI showed a hypointense tumor on T1-weighted image and a hyperintense tumor on T2-weighted image. A dynamic gadolinium ethoxybenzyl diethylenetriamine pentaacetic acid (Gd-EOB-DTPA)-enhanced MRI study showed an internal heterogeneous mass. T2-weighted MRI showed the mass as hypointense in relation to cerebrospinal fluid. Although the final diagnosis of HSH was made histopathologically, understanding of its radiologic appearance is important to avoid unnecessary surgery, and HSH should be included in the differential diagnoses of a hepatic lesion with delayed enhancement [17]. In the present case, the main tumor was shown as a slightly hyperintense area on diffusion-weighted MRI image. Hida *et al*. [18] reported that HSH had a high apparent diffusion coefficient (ADC) on MRI. Therefore, HSH cannot be differentiated from hepatic metastasis and cholangiocellular carcinoma based on the MRI findings. The presence of many hyalinized tissues with poor cellular and fibrous components as revealed microscopically might be a cause of the high ADC value. Based on the radiologic findings and due to a suspicion of carcinomas, we performed a segmentectomy.

Electron microscopy showed a hyalinized lesion, with cells resembling histiocytes and fibroblasts. In a previous study, the number of mast cells correlated significantly with vascular proliferation and correlated inversely with the degree of fibrosis [9]. Gross and Wolbach [19] were the first to describe sclerosing hemangioma as having endothelial origin. They recognized a spectrum of histological changes from the early overgrowth of fibrous tissue in a hemangioma to the complete replacement of hemangiomatous structures by fibrosis and accumulation of lipid and hemosiderin-laden histiocytes. These changes are thought to represent regressive phenomena that occur in varying degrees leading eventually to a number of different patterns.

Immunohistochemical, electron microscopic, and various molecular pathological techniques have been utilized to diagnose pulmonary sclerosing hemangioma [20]. The cause of the sclerosed process, which eventually destroys the blood vessels, remains unknown. Whether the stimulus leading to sclerosis is initiated by the endothelial cells or by the surrounding fibroblasts is uncertain. In view of the fact that endothelial cells may act as facultative fibroblasts and become ultrastructurally similar to fibroblasts, we favor the former. Our immunohistochemical staining for caveolin-1 and electron microscopic findings shed new light on the process of dysregulated angiogenesis in this very rare disorder. In electron microscopic study, we showed that LCH are composed of numerous caverns in various shapes and sizes. The caverns form a labyrinth, communicating with each other. They are lined by spindle-shaped endothelial cells containing multiple micropinocytic vesicles or caveolae along the luminal surface. Caveolin-1 remains overexpressed in the endothelial cells of the capillary tufts at the edge of the hemangioma but is reduced in the sclerosed hyaluronic lesion (Figure 6a and b). Hemangioma has been reported to be composed of numerous caves forming a labyrinth with narrow interconnecting canals and lined by spindle-shaped endothelial cells with multilayered smooth muscle cells [21,22]. We demonstrated endothelial cells together with multilayered smooth muscle cells by electron microscopy and the difference between high expression of caveolin-1 in the endothelial cells and scanty expression of fibroblasts by immunohistochemistry. Caveolin-1 inhibits vascular smooth muscle cell (VSMC) proliferation in part by modulating key cycle-regulatory proteins. Furthermore, overexpression of caveolin-1 has a dramatic effect on VSMC response to growth stimuli, including induction of apoptosis [23]. Caveolin-1 immunostaining of endothelium showed increased punctuate caveolin-1 reactivity on a few or none of the pericytes [24]. Caveolin-1 expression has been described in a range of vascular neoplasms [14], and caveolin-1 in endothelial cells regulates microvascular permeability [10]. In our ultrastructural findings, numerous caveolae were found in cavernous hepatic endothelial cells of the residual cavernous hemangioma. By contrast, a few caveolae were found in the remnant capillary endothelial cells of HSH. Functionally, caveolae of cavernous hepatic endothelial cells can interact with numerous kinds of extracellular matrix molecules and facilitate angiogenesis [25].

## Conclusions

HSH is a rare condition. Comparison of radiological findings of the lesion over a period of 10 years was valuable in providing insight for the evolutional process from cavernous hemangioma to sclerosed hemangioma.

## Consent

Written informed consent was obtained from the patient for publication of this case report and any accompanying images. A copy of the written consent is available for review by the Editor-in-Chief of this journal.

## Competing interests

The authors declare that they have no competing interests.

## Authors’ contributions

YT performed the surgery. YS and MW collected the references and contributed to the writing. HI and HT reviewed CT and MRI. HYa interpreted the liver histology and contributed to the writing. HYo and YS wrote the paper. All authors have read and approved the final manuscript.

## Supplementary Material

Additional file 1 Supplemental data. Click here for file
