# Supplementary material for: A hepatic sclerosed hemangioma with significant morphological change over a period of 10 years: a case report
Source: J Med Case Rep. 2013 May 28;7:139. doi: 10.1186/1752-1947-7-139 (PMC3750244; doi:10.1186/1752-1947-7-139)
Supplement: Additional file 1 — Supplemental data. [file 1752-1947-7-139-S1.doc]

**Supplemental data**

**Electron microscopy**

Frozen tissue blocks were stored at –80°C for up to 8 weeks. The blocks were brought directly from storage at –80°C into a fixative of 2.5% glutaraldehyde in 0.1-M phosphate buffer (pH 7.4) at 4°C [1]. Fixation was performed for 48 hours at 4°C under continuous rotation of the vials. After rinsing overnight in the same buffer at room temperature, the tissue blocks were postfixed in 1% osmium tetroxide for 1 hour in the dark at room temperature. Dehydration and embedding in epoxy resin were conducted according to routine procedures. These sections were stained with toluidine blue to select an area for ultra-thin sectioning. After embedding in Epon, ultra-thin sections were cut using a diamond knife on a LKB ultramicrotome. They were stained with uranyl acetate and observed under a transmission electron microscope (JEM-1200 EX; JEOL Tokyo, Japan) with 80-kV acceleration voltage.

# **Immunohistochemistry**

Resected liver tissues (approximately 4cm × 3cm × 1.5cm) were fixed in formalin and embedded in paraffin. First, 4mm sections were cut from the paraffin blocks, deparaffinized with xylene, and dehydrated using graded ethanol. They were incubated overnight at 4°C with 1:200 dilution of rabbit anti-caveolin-1 polyclonal antibodies (sc-894; Santa Cruz Biotechnology). Then the sections were incubated with EnVision™ reagents (Dako Inc., Tokyo, Japan) at room temperature for 30 minutes. After repeated washing with phosphate-buffered saline, the sections were reacted with diaminobenzidine containing 0.01% hydrogen peroxide, and counterstained with hematoxylin for light microscopic study [2].

**References**

1. [Vogels IM](http://www.ncbi.nlm.nih.gov/pubmed?term=Vogels IM%5BAuthor%5D&cauthor=true&cauthor_uid=8256298), [Van Noorden CJ](http://www.ncbi.nlm.nih.gov/pubmed?term=Van Noorden CJ%5BAuthor%5D&cauthor=true&cauthor_uid=8256298), [Hoeben KA](http://www.ncbi.nlm.nih.gov/pubmed?term=Hoeben KA%5BAuthor%5D&cauthor=true&cauthor_uid=8256298), [Korper W](http://www.ncbi.nlm.nih.gov/pubmed?term=Korper W%5BAuthor%5D&cauthor=true&cauthor_uid=8256298), [Jonges GN](http://www.ncbi.nlm.nih.gov/pubmed?term=Jonges GN%5BAuthor%5D&cauthor=true&cauthor_uid=8256298), [Everts V](http://www.ncbi.nlm.nih.gov/pubmed?term=Everts V%5BAuthor%5D&cauthor=true&cauthor_uid=8256298). **Use of frozen biologic material for combined light and electron microscopy.** [*Ultrastruct Pathol*.](http://www.ncbi.nlm.nih.gov/pubmed/8256298) 1993, **17**:537–546.

2. Yokomori H, Oda M, Yoshimura K, Kaneko F, Hibi T. [**Aquaporin-1 associated with hepatic arterial capillary proliferation on hepatic sinusoid in human cirrhotic liver.**](http://www.ncbi.nlm.nih.gov/pubmed/22093331) *Liver Int*. 2011, **31**:1554–1564.
